# The Protective Effect and Mechanism of a Phytochemical Extract from the Wild Vegetable Shutou (*Crateva unilocularis* Buch.) against Acetaminophen-Induced Liver Injury in Mice

**DOI:** 10.3390/foods12163109

**Published:** 2023-08-18

**Authors:** Meimei Shan, Qian Ma, Yilin Sun, Fengyi Gao, Shengbao Cai

**Affiliations:** 1 Faculty of Food Science and Engineering, Kunming University of Science and Technology, Kunming 650500, China; 18239031665@163.com (M.S.); kmustma95@163.com (Q.M.); syl115630519@163.com (Y.S.); 2 College of Biology and Food, Shangqiu Normal University, Shangqiu 476000, China; 3 Department of Food Science, Northeast Agricultural University, Harbin 150030, China

**Keywords:** APAP, *Crateva unilocularis* Buch., liver damage, reactive oxygen species

## Abstract

Acetaminophen (APAP) abuse is a common public health problem which can cause severe liver damage. However, strategies for dealing with this situation safely and effectively are very limited. The goal of the current work was to evaluate the protection and potential molecular mechanisms of an ethanol extract from shoots of the wild vegetable shutou (*Crateva unilocularis* Buch.) (ECS) against APAP-induced liver damage in mice. Mice orally received ECS for seven days (300 or 600 mg/kg b.w. per day) before being intraperitoneally injected with APAP (250 mg/kg). Results exhibited that ECS obviously decreased the content of alkaline phosphatase, alanine aminotransferase, aspartate transaminase, and malondialdehyde (*p* < 0.05). Catalase and superoxide dismutase were notably restored (*p* < 0.05), and the content of reduced glutathione was obviously increased (*p* < 0.05). Moreover, ECS significantly inhibited the secretion of interleukin-1β and tumor necrosis factor-α (*p* < 0.05). Further analyses of the mechanisms showed that ECS may alleviate oxidative stress in the liver by increasing the expression of the nuclear factor erythroid-2-related factor 2 and NADH quinone oxidoreductase 1 proteins, and may suppress liver inflammation by inhibiting the expression of the phosphorylated-inhibitor kappa B alpha/inhibitor kappa B alpha, phosphorylated-nuclear factor κB/nuclear factor κB, and cyclooxygenase-2 proteins. Meanwhile, ECS inhibited hepatocyte apoptosis by enhancing B-cell lymphoma gene 2 and suppressing Bcl-2-associated X protein. In summary, ECS may be used as a dietary supplement to prevent the liver damage caused by APAP abuse.

## 1. Introduction

The liver, which is responsible for the metabolism of nutrients and detoxification, is a vulnerable organ [1]. Liver-related disorders such as hepatitis, cirrhosis, and liver cancer have been identified as a major public health concern worldwide [2]. The Global Burden of Disease Study states that liver-related diseases caused approximately 226.4 million deaths in 195 regions worldwide [3]. Drug-induced liver injury (DILI) can result in liver failure and possibly death [4], which is the most prevalent reason for acute liver failure in most Western nations, accounting for more than half of all cases. Furthermore, DILI-related hospital admissions account for 5% of total hospital admissions [5,6]. It has been reported that acetaminophen (APAP) overdose is the most common reason for DILI [7]. As a classic antipyretic and analgesic, it is common to use APAP as a treatment for colds and fevers. APAP is safe and effective when it is within the therapeutic dosage range [8]. However, excessive use of APAP can result in liver damage, and cases of APAP-induced hepatotoxicity are responsible for about 50% of acute liver injury (ALI) cases in developed countries [9]. Thus far, N-acetylcysteine (NAC) has exclusively been applied as an clinical treatment for liver injury caused by APAP; however, NAC is only effective in the early stages of ALI, and can cause side effects such as vomiting, headaches, and nausea [10,11]. Therefore, more potent and less side-effect-prone methods are needed to address this problem.

At the recommended dose, 85–90% of APAP is excreted in the urine after metabolism by glucuronidation or sulfation; less than 10% of APAP is excreted by the cytochrome p450 (CYP450) into n-acetyl-p-benzoquinone imine (NAPQI), which is rapidly converted into nontoxic metabolites by glutathione (GSH) [12]. However, APAP overdose leads to excess production of NAPQI, which depletes intracellular GSH, leading to over-accumulation of reactive oxygen species (ROS) and induction of an oxidative stress state, eventually leading to the death of hepatocytes and even liver failure [13,14]. As an important antioxidant sensor, nuclear factor erythroid-2-related factor 2 (Nrf2) performs a key function in enhancing cells’ initial defense against free radicals by regulating adaptive antioxidant responses in the body [12]. In several studies, natural plant active ingredients such as farrerol and licochalcone A have been shown to trigger the Nrf2 signaling pathway, leading to increased expression of Phase II detoxification/antioxidant enzymes, including NADH quinone oxidoreductase 1 (NQO1), superoxide dismutase (SOD), and catalase (CAT) [15], in addition to participating in antioxidant responses [16,17]. Moreover, the hepatotoxicity caused by APAP stimulates the hepatic macrophages to release various pro-inflammatory cytokines, e.g., interleukin-1β (IL-1β) and tumor necrosis factor-α (TNF-α) [18], which enables the nuclear factor κB (NF-κB) signaling pathway to exacerbate the inflammatory response [2]. In addition, the apoptosis of hepatocytes participates in the liver injury process induced by APAP [19,20]. It has been shown that the phosphatidylinositol 3-kinase (PI3K)/protein kinase B (Akt) signaling pathway is a crucial target for protecting the liver by inhibiting apoptosis under oxidative stress and inflammatory conditions [21]. Akt is a key downstream target of PI3K, and activated Akt can further regulate its downstream regulatory factors, such as Bcl-2-associated X protein (Bax) and B-cell lymphoma gene 2 (Bcl-2), to inhibit apoptosis [22]. Bcl-2 is an important regulator of mitochondrially mediated apoptosis, and acts as a crucial role in the apoptotic pathway to inhibit apoptosis by preventing depolarization of the mitochondrial membrane [2]. As a member of the Bcl-2 family, Bax can interact with and inactivate Bcl-2 to enhance apoptosis [23]. Ding et al. showed that the natural active ingredient gambogenic acid can exhibit antiapoptotic activity by activating the signaling pathway of PI3K/Akt [24].

The wild vegetable shutou, shown in Figure S1, is the shoot of *Crateva unilocularis* Buch., and has great nutritional value. It is primarily found in China, India, Vietnam, and other countries. Based on folk records, the wild vegetable shutou has medicinal properties as an anti-inflammatory and for detoxification [25]. Previous studies have shown that the wild vegetable shutou mainly contains polyphenols and saponins, and can scavenge oxygen-free radicals effectively [26]. However, there are no relevant studies on its preventive effects and its underlying mechanisms in acute liver injury from APAP, which may be detrimental to its further development and utilization. Therefore, in this study APAP was selected to establish a model to determine the prevention and potential mechanisms of the wild vegetable shutou against ALI, which may provide theoretical guidance for the further utilization of shutou and offer an alternative and safe strategy to deal with liver injury from APAP abuse.

## 2. Materials and Methods

### 2.1. Chemicals

APAP with a purity greater than 98% was bought from Sigma-Aldrich (St. Louis, MO, USA). All standards (purity ≥ 98%) used for the quantitative analysis were bought from Chengdu Must Bio-Technology Company (Chengdu, China). CAT, SOD, GSH, malondialdehyde (MDA), aspartate transaminase (AST), alkaline phosphatase (ALP), and alanine aminotransferase (ALT) biochemical kits were purchased from Jiancheng Bioengineering Institute (Nanjing, China). TNF-α and IL-1β enzyme-linked immunosorbent test (ELISA) kits were supplied by Multi Sciences Biotech Co. (Hangzhou, China). A kit for the extraction of nuclear and cytoplasmic proteins, along with a lysis buffer that contains the inhibitors of proteases, was supplied by the Beyotime company (Shanghai, China). A BCA kit was obtained from BioSharp (Hefei, China). Tris-buffered saline powder (TBS) was supplied by Wuhan Xavier Biotechnology Co. Ltd. (Wuhan, China). Antibodies, including Nrf2, NQO1, NF-κB, phosphorylated-nuclear factor κB (*p*-NF-κB), cyclooxygenase-2 (COX-2), phosphorylated-phosphatidylinositol 3-kinase (*p*-PI3K) (phospho-PI3K Y467/Y199/Y464), PI3K, phosphorylated-protein kinase B (*p*-Akt) (Ser 473), Akt, Bax, and Bcl-2 were supplied by ABclonal company in Wuhan, China.

### 2.2. Extraction and Analysis of Samples 

Samples of the wild vegetable shutou were purchased from Yangbi County, Dali City, Yunnan, China (2019), and were identified by Prof. Jianxin Cao of Kunming University of Science and Technology. The samples were preserved at the Faculty of Food Science and Engineering of Kunming University of Science and Technology. The fresh wild vegetable shutou was lyophilized and powdered to pass through a sieve (60-mesh). Then, the ultrasonic extraction method with 80% ethanol of the dried sample powder was used to obtain an ethanol extract of the wild vegetable shutou (ECS). Lyophilization of ECS and qualitative and quantitative analysis of the chemicals in the ECS by UHPLC-ESI-HRMS/MS was the same as in a previous report [26]. Lyophilized powder of the extract was used for the subsequent animal experiment. 

### 2.3. Animal Experiments

**Figure 1:**
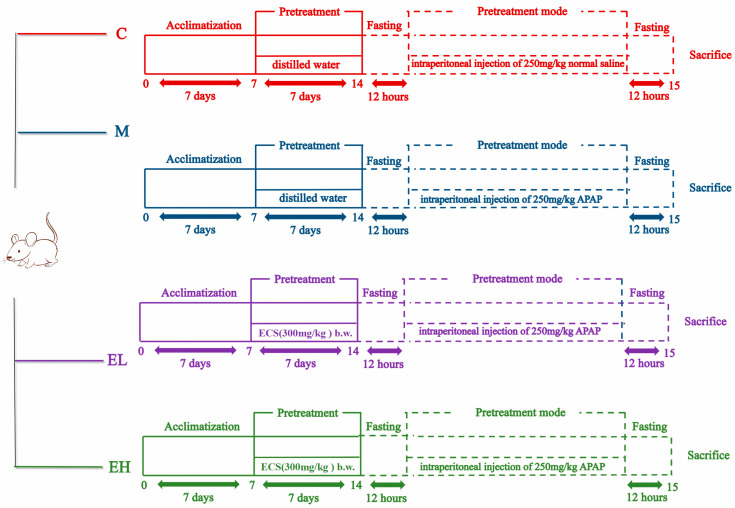
Outline of the experiment on the effect of ECS on ALI caused by APAP. C, M, EL, and EH represent the control, model, low-dose, and high-dose ECS groups (300 or 600 mg kg^−1^ of b.w.), respectively.

In total, 40 male mice (Kunming species, 20–22 g, SPF grade) were bought from Hunan SJA Laboratory Animal Company (Hunan, China; Certificate No. SCXK (Xiang) 2019-0004) and housed under standard conditions (23 °C ± 2 °C, 40–75% humidity, and a light/dark cycle of 12 h) and fed with maintaining feedstuffs (fiber, 5%; fat, 5%; protein, 20%; carbohydrates, 60%) provided by Kunming Medical University (Kunming, China). Figure 1 depicts a schematic of the design of the animal assay. Mice were assigned randomly to the following groups one week after acclimatization: C (Control), M (Model), EL (low-dose), and EH (high-dose) groups. The EL and EH groups received 300 mg or 600 mg of ECS per kilogram of body weight (b.w.) per mouse, respectively. The powdered extract was weighed and ultrasonically redissolved by distilled water, and mice in the sample intervention groups (Groups EL and EH) were administered the extract by gavage once a day based on a preliminary experiment. Distilled water was provided to Groups C and M in the specified quantities. After one week of continual administration, all mice fasted for 12 h. Then, APAP (250 mg kg^−1^), dissolved in a carboxymethyl cellulose sodium salt (CMC-Na) solution was intraperitoneally injected into Groups M, EL, and EH. An intraperitoneal injection of a CMC-Na solution was conducted to mice of Group C. All mice were euthanized with 2% isoflurane after 12 h. Using heparin sodium as an anticoagulant, blood of each mouse was collected by eyeball extirpating and centrifuged at 1800× *g* (4 °C) for five minutes to obtain plasma. All animal plasma samples were stored at −80 °C to facilitate further analysis. After the livers of the mice were weighed, the liver lobules were removed and fixed in a paraformaldehyde solution (4%) to perform histological examination. After permeating the liver tissues with liquid nitrogen, each sample was placed at −80 °C. The animal experiments were strictly carried out according to the Guidelines for the Care and Use of Experimental Animals, and were approved by the Animal Experiment Ethics Committee of Shangqiu Normal University (No. 2022-0312). 

### 2.4. Evaluation of Biochemical Markers in the Liver and Plasma

The livers were prepared as a tissue homogenate according to a previously reported method [27]. The enzyme activities of ALT, AST, and ALP in the plasma and those of SOD, CAT, MDA, GSH, TNF-α, and IL-1β in the homogenate of liver were determined (*n* = 10) based on the kits’ instructions. 

### 2.5. Histopathological Evaluation

The histopathology of the hepatic lobules (*n* = 4) was assessed by hematoxylin–eosin (H&E) staining. A 4% paraformaldehyde solution was used to fix the hepatic lobules, then the liver lobules were dehydrated, soaked, and embedded in paraffin wax. Each section was sliced at a thickness of 5 mm. H&E staining was carried out on sections of the liver lobes, which were observed with a microscope (Olympus IX83, Tokyo, Japan). 

### 2.6. Immunohistochemical Evaluation

Liver segments (*n* = 4) from each group were dewaxed and rehydrated with alcohol before being incubated for 30 min with 5% bovine serum protein, held with the primary antibody overnight at 4 °C (*p*-NF-κB), and washed three times in PBS (PH = 7.4) for 5 min each. Anti-rabbit IgG secondary antibody was then incubated at room temperature away from the light for 10 min. Finally, the nuclei were stained by DAPI for 10 min at ambient temperature away from the light and each section was measured with a microscope.

### 2.7. Immunofluorescence

Immunofluorescence was used to detect Nrf2 expression in the nucleus. After a liver section (*n* = 4) had been obtained according to the procedure described above, it was sealed with 5% BSA solution (30 min) and reacted with primary antibody overnight. Afterwards, it was washed with PBS three times and combined with the secondary antibody of cyanine dye-combined anti-rabbit IgG. The cell nuclei of the liver slices were stained by DAPI and observed with a microscope.

### 2.8. Western Blotting 

The method of Zhou et al. [27] was used to detect the target proteins in the liver tissues (*n* = 4). Briefly, a certain weight (100 mg) of liver tissue was weighed from each mouse and mixed proportionally with a lysis buffer containing protease and phosphatase inhibitors. A Scientz-ii D ultrasonic cell shredder was applied to homogenize the tissue (Ningbo, China). The tissue was cleaved under an ice bath condition and then centrifuged at 10,000× *g* for 5 min at 4 °C. The supernatant was gathered and the total protein amount in the supernatant was determined by a BCA kit. The protein was isolated by SDS-PAGE gel electrophoresis. The 10% gels were configured according to the kit’s instruction (Wuhan Xavier Biotechnology Co., Ltd., Wuhan, China). A total of 30 μg of each sample was first loaded into the SDS-PAGE gel, then isolated by electrophoresis. A nitro-fiber (NC) membrane was used for the target protein transfer. The NC membrane was first blocked in 5% skim milk powder for 1 h at room temperature and then incubated overnight with the primary antibody at 4 °C, followed by three rinses with a TBS-Tween solution (TBST) and incubation with the secondary antibody for 1 h at room temperature. Eventually, the expression levels of protein were determined using a chemiluminescence detection reagent (G2014, Wuhan Xavier Biotechnology Co. Ltd., Wuhan, China) in an imaging system (VILBER Fusion FX7, VILBER Lourmat, Marne-la-Vallee, France).

### 2.9. Statistical Analysis

Data are presented as the mean ± standard error (S.E.). OriginLab 8.5 software (Northampton, MA, USA) was used for one-way ANOVA and significant difference (Tukey’s test) analyses. 

## 3. Results and Discussion

### 3.1. Qualitative and Quantitative Results of UHPLC-ESI-HRMS/MS for ECS

The qualitative and quantitative results of the chemical substances in the ECS are shown in Table S1 and Figure S2. In total, thirteen compounds were identified, including six saponins, six phenolics, and one organic acid (quinic acid). The quantitative results showed that saponins were the main substances, with ginsenoside Ro being the most abundant, followed by Chikusetsusaponin IVa. This is generally consistent with a previous report [26] in which fourteen compounds were detected; saponin 3-O-β-D-Glc-(1→2)-[[2-carboxy-1-(carboxymethyl)-2-hydroxyethyl]-(1→3)]-β-D-GluA-28-O-β-D-Glc-oleanolic acid, with the lowest content, was not detected in this study. The slight difference in the composition of the components and contents may be because the samples came from different batches, although the samples of wild shutou used in this study were purchased from the same region as those in the previous study [26].

### 3.2. ECS Improved the Liver Coefficient 

**Figure 2:**
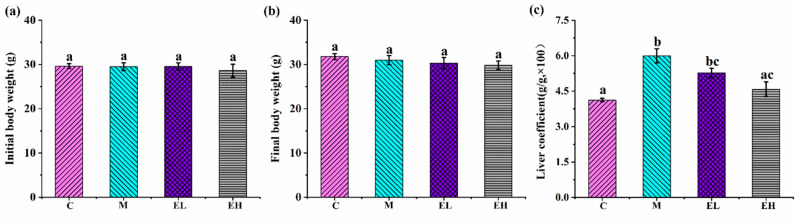
Influence of ECS on the body weight and liver coefficient in mice with APAP-induced ALI: (**a**,**b**) initial and final body weight, respectively, and (**c**) liver coefficient. Data are presented as the mean ± S.E. (*n* = 10). For each biochemical indicator, different letters suggest significant differences (*p* < 0.05). C, M, EL, and EH represent control, model, low-dose, and high-dose ECS groups (300 or 600 mg kg^−1^ of b.w.), respectively.

It has been reported that APAP damage in the liver can cause an increase in the liver coefficient, and that this can be reduced by quercitrin extracted from Acacia catechu [28,29]. As shown in Figure 2, both the liver coefficient and the body weight of the four groups were compared. While APAP treatment did not affect body weight (*p* > 0.05), it was observed to considerably increase the liver coefficient (*p* < 0.05). The two dose-treated groups exhibited no remarkable changes in body weight when compared with the model group (Figure 2a,b). For the increased liver coefficient caused by APAP, the change in the EL group after ECS treatment was insignificant, but while that in the EH group was noticeably lower in comparison that in Group M (*p* < 0.05). These results suggest that higher concentrations of ECS treatment attenuated the increase in the liver coefficient induced by APAP injury.

### 3.3. ECS Protected the Liver against APAP-Induced Injury

**Figure 3:**
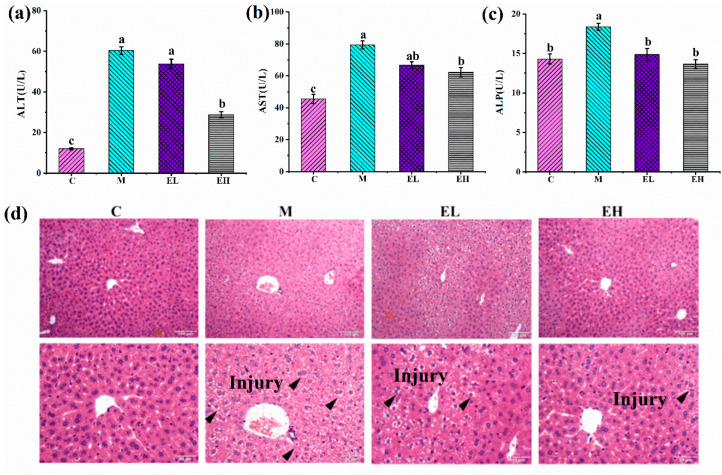
Effects of ECS on (**a**) ALT, (**b**) AST, and (**c**) ALP in the plasma along with (**d**) liver histopathological changes as observed by H&E staining. Data are presented as the mean ± S.E. (*n* = 10). For each biochemical indicator, different letters suggest significant differences (*p* < 0.05). ALP, ALT, and AST represent alkaline phosphatase, alanine, aminotransferase, and aspartate transaminase, respectively, while C, M, EL, and EH represent the control, model, low-dose, and high-dose ECS groups (300 or 600 mg kg^−1^ of b.w.), respectively.

Liver damage can be assessed by measuring the specific enzymes’ activities in the plasma. ALT, AST, and ALP are commonly used to assess the liver toxicity caused by APAP [30,31]. As shown in Figure 3, when comparing Group M to Group C, the levels of the above indices were statistically higher (*p* < 0.05). However, in comparison with Group M, ALT and AST in the EH group significantly declined (*p* < 0.05). Notably, the ALP levels in both the EL and EH groups were considerably lower than in Group M (*p* < 0.05), and were almost similar to those in Group C (*p* > 0.05). Additionally, H&E staining indicated that the liver tissues in Group C were normal and intact (Figure 3d). Comparatively, the livers in Group M were severely injured, including central lobular necrosis, hepatic cell edema and degeneration, central venous endothelial destruction, nucleolysis, pyrosis, and nuclear rupture. ECS treatment reduced the severity of liver injury with a dose-dependent effect, especially in the livers of the EH group, which were approximately similar to those of Group C. 

Excessive intake of APAP can lead to severe liver injury. The most obvious manifestation is an increase in the biochemical parameters of liver injury (ALT, AST, and ALP) in the plasma and damage to the integrity of liver cells. In the high-dose ECS group, the content of ALT, AST, and ALP decreased significantly in the plasma. Meanwhile, the histopathological results showed that the structural damage of the hepatocytes was reduced in the ECS-treated groups. These results suggested that ECS protects mice from acute liver injury induced by APAP.

### 3.4. ECS Alleviated APAP-Induced Oxidative Stress in the Liver Tissue

**Figure 4:**
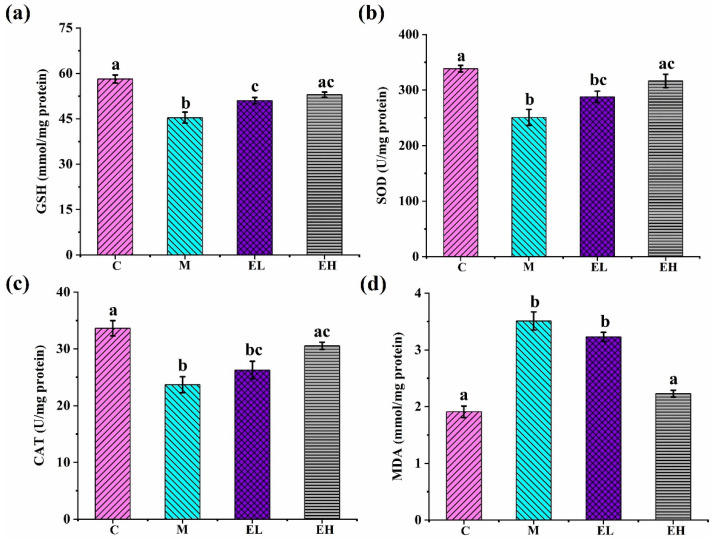
The effects of ECS on (**a**) GSH, (**b**) SOD, (**c**) CAT, and (**d**) MDA in the liver tissue. Data are presented as the mean ± S.E. (*n* = 10). For each biochemical indicator, different letters suggest significant differences (*p* < 0.05). SOD, superoxide dismutase; GSH, reduced glutathione; CAT, catalase; MDA, malondialdehyde; C, M, EL, and EH represent control, model, low-dose, and high-dose ECS groups (300 or 600 mg kg^−1^ of b.w.), respectively.

When APAP is overused, GSH becomes depleted, leading to the accumulation of ROS and subsequent mitochondrial oxidative stress and dysfunction [32]. Increased lipid peroxidation and a decrease in the function of the (non)-enzymatic antioxidant defense systems occur in acute liver injury caused by APAP [33]. GSH is a key antioxidant for scavenging NAPQI, ROS, and peroxynitrite in APAP-related hepatotoxicity [34]. As shown in Figure 4, the level of GSH (Figure 4a) in Group M was substantially lower compared with that in Group C (*p* < 0.05). Oxidative stress is greatly influenced by antioxidant enzymes, including SOD and CAT. SOD and CAT in Group M were noticeably lower when compared with those in Group C (*p* < 0.05), while the SOD, CAT, and GSH levels in Group EH were remarkably higher than their counterparts in Group M (*p* < 0.05). The accumulation of ROS is harmful to the cells, causing lipid oxidation and ultimately cell damage. MDA is generally a characteristic parameter of the lipid peroxidation degree [35]. As illustrated in Figure 4d, Group M had a noticeably higher MDA than Group C (*p* < 0.05), while Group EH had a significantly lower MDA content than Group M (*p* < 0.05). The buildup of free radicals, which results in oxidative stress and consequent liver damage, is a key mechanism of APAP-induced liver damage [36]. Studies have shown that excessive intake of APAP reduces the activities of antioxidant enzymes and leads to the accumulation of MDA [37,38]. Our results confirm that APAP reduced GSH, CAT, and SOD and increased MDA in liver tissues, suggesting that redox homeostasis was disrupted in the liver tissues and lipid peroxidation occurred. After ECS treatment, especially in the mice in the EH group, CAT and SOD in the liver tissues were restored and GSH and MDA almost returned to normal. These results show that ECS may be able to deal with the oxidative stress and damage caused by APAP through regulating the activity of antioxidant enzymes and restoring MDA. 

The first line of defense against over-production of ROS in the body consists of antioxidant enzymes such as SOD and CAT. When they are damaged or depleted, signals trigger the activation of specific proteins that regulate oxidative stress [39]. Nrf2 not only regulates liver detoxification, it regulates the expression of the oxidative stress kinase, thereby playing a pivotal function in oxidative stress in the liver. For liver illnesses caused by APAP, such as ALI, the Keap1-Nrf2 pathway has been identified as a possible therapeutic target [40]. Under nonstressed conditions, Keap1 and Nrf2 are tightly bound in the cytoplasm; however, when triggered by ROS or electrophiles, Nrf2 separates from Keap1 and translocates to the nucleus after gathering in the cytoplasm, where it recognizes and reacts to the antioxidant response element (ARE) to activate the antioxidant enzyme (NQO1) downstream [41,42,43].

**Figure 5:**
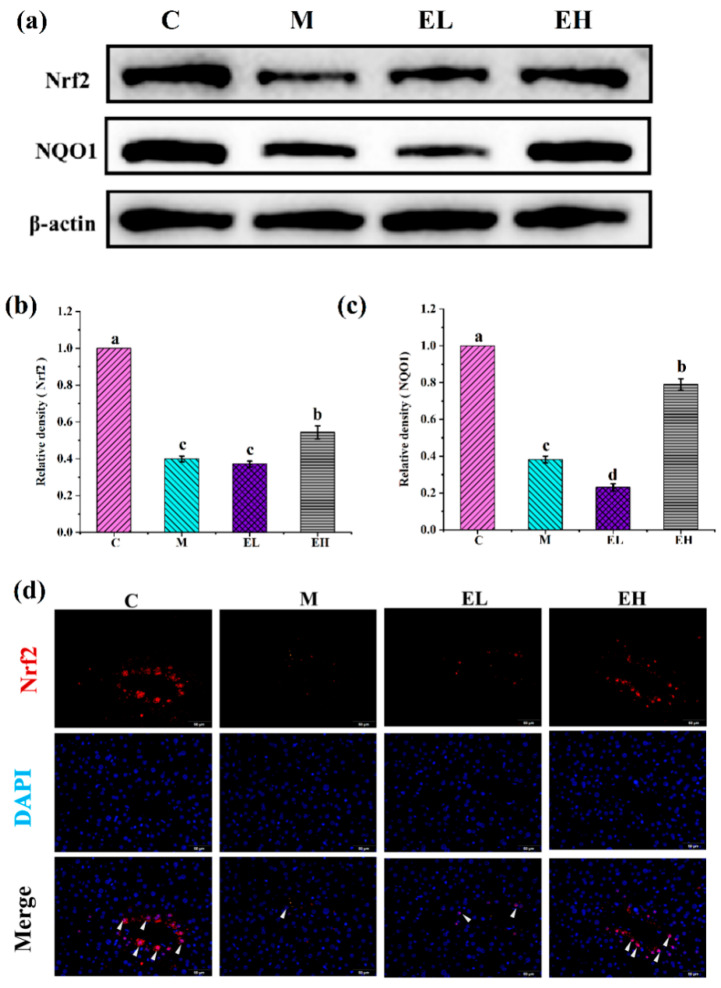
Effects of ECS on the Nrf2 and NQO1 proteins and immunofluorescence of the nuclear transfer of Nrf2 in the livers of mice with or without APAP treatment. (**a**) Immunoblotting analysis of the Nrf2 and NQO1 proteins. The relative contents of (**b**) Nrf2 and (**c**) NQO1 normalized with β-actin and Group C, expressed by the grayscale. (**d**) The Nrf2 immunofluorescence results entering the nucleus. Data are presented as the mean ± S.E. (*n* = 4). For each protein, different letters suggest significant differences (*p* < 0.05). Nrf2 and NQO1 represent nuclear factor erythroid-2 related factor 2 and NADH quinone oxidoreductase 1, respectively; C, M, EL, and EH represent control, model, low-dose, and high-dose ECS groups (300 or 600 mg kg^−1^ of b.w.), respectively.

The protein expression associated with oxidative stress (Nrf2 and NQO1) is illustrated in Figure 5. When compared with Group C, the aforementioned important proteins were noticeably downregulated in Group M (*p* < 0.05). Nrf2 (Figure 5a,b) did not increase in Group EL compared with Group M; in group EH, however, the expression of this protein was remarkably upregulated (*p* < 0.05). Similarly, the expression of NQO1 (Figure 5a,c) was significantly increased after administration of a high dose of ECS (EH group) in comparison with Group M (*p* < 0.05). Meanwhile, the Nrf2 expression in the nucleus of hepatocytes was visually observed through immunofluorescence analysis (Figure 5d). In comparing Group M and Group C, a lower content of Nrf2 protein entered the nucleus. Specifically, treatment with 600 mg kg^−1^ of ECS (EH group) could stimulate the nuclear entry of Nrf2, potentially contributing to the levels of the second-generation antioxidant enzymes and alleviating liver injury induced by APAP [28]. The results of Western blotting and immunofluorescence showed that ECS may activate Nrf2 and increase its nuclear expression, thereby increasing the expression of the downstream protein NQO1, which has an antioxidant effect. Studies have shown that natural plant active ingredients attenuate oxidative stress and protect the hepatocytes from oxidative stress injury through the Nrf2/Keap1 system. For example, baicalein and baicalin reduced APAP-induced hepatotoxicity by blocking the binding of Nrf2 to Keap1 [44]. In these results, the expression of Nrf2 and its downstream molecule NQO1 were measured, and the Nrf2-mediated signal pathway was found to be suppressed in APAP-induced liver injury. ECS showed a protective effect by enhancing the expression of Nrf2 and NQO1. This implies that ECS has antioxidant effects on liver injury caused by APAP by triggering the Nrf2-mediated pathway.

### 3.5. ECS Reduced the APAP-Induced Inflammatory Response in the Liver

**Figure 6:**
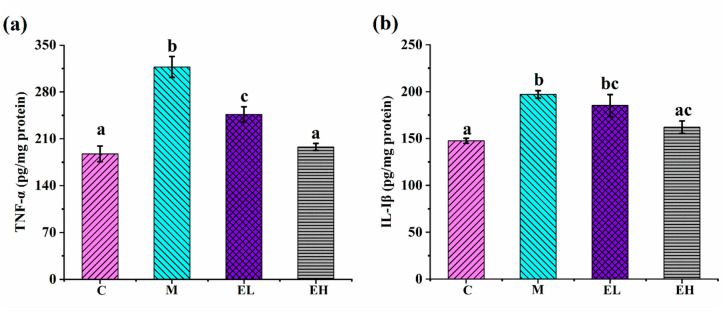
Effects of ECS on (**a**) TNF-α and (**b**) IL-1β in the liver tissue. Data are presented as the mean ± S.E. (*n* = 10). For each biochemical indicator, different letters indicate significant differences (*p* < 0.05). TNF-α, tumor necrosis factor-α; IL-1β, interleukin-1β; C, M, EL, and EH represent control, model, low-dose, and high-dose ECS groups (300 or 600 mg kg^−1^ of b.w.), respectively.

APAP overdose-induced hepatotoxicity is closely related to liver inflammation [45]. Studies have shown that APAP can lead to cellular inflammation by modulating several inflammatory mediators, such as the proinflammatory cytokines TNF-α and IL-1β [46,47]. As presented in Figure 6, in comparison with Group C, the contents of TNF-α and IL-1β in group M increased markedly (*p* < 0.05), indicating the occurrence of inflammation and that APAP caused an increase in inflammatory factors, which is consistent with previous results [47]. In comparison with Group M, TNF-α in the EH and EL groups was markedly reduced (*p* < 0.05) and both the TNF-α and IL-1β levels in the EH group were markedly decreased to a level similar to Group C (*p* > 0.05). Thus, ECS reversed the APAP-induced increase in proinflammatory cytokines in the liver, including TNF-α and IL-1β. 

**Figure 7:**
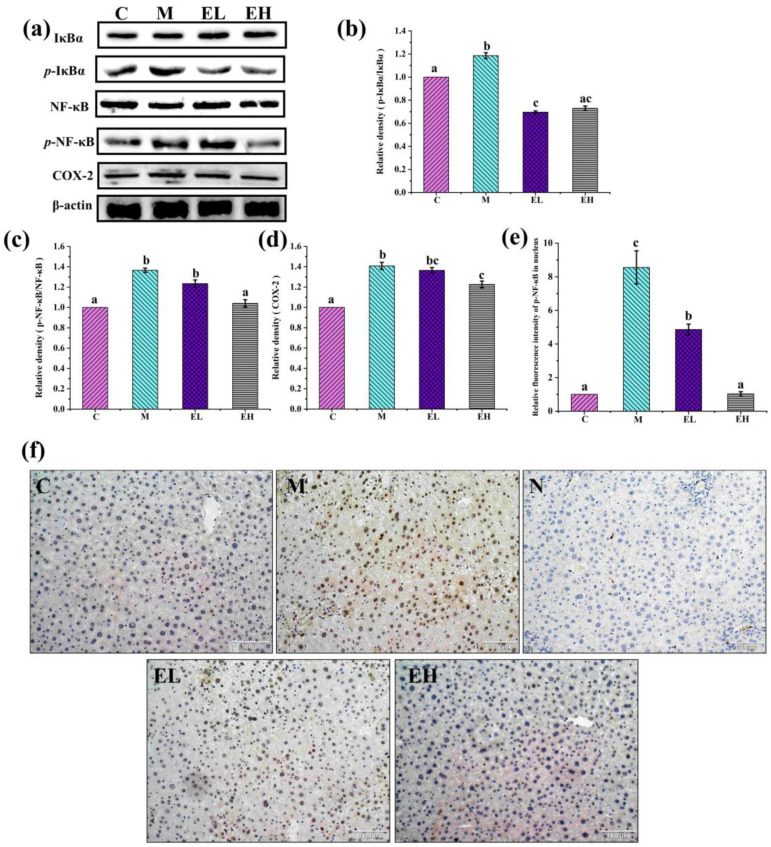
Effects of ECS on the contents of several critical proteins involved in the inflammation signaling pathway. (**a**) Immunoblotting of *p*-IκBα/IκBα, *p*-NF-κB/NF-κB, and COX-2 proteins. (**b**–**d**) Relative expression of *p*-IκBα/IκBα, *p*-NF-κB/NF-κB, and COX-2 proteins, respectively. The relative protein expressions were normalized with β-actin and Group C, shown as grayscale. (**e**,**f**) The immunohistochemical results of N-NF-κB entering into the nucleus. Data are presented as the mean ± S.E. (*n* = 4). For each protein, significant differences are marked by different letters (*p* < 0.05). *p*-IκBα/IκBα, *p*-NF-κB/NF-κB, COX-2, and N-NF-κB represent phosphorylated-inhibitor kappa B alpha/inhibitor kappa B alpha, phosphorylated-nuclear factor κB/nuclear factor κB, cyclooxygenase-2, and nuclear-nuclear factor κB, respectively; C, M, EL, and EH represent control, model, low-dose, and high-dose ECS groups (300 or 600 mg kg^−1^ of b.w.), respectively.

NF-κB is a crucial regulator of the hepatic inflammatory signaling pathways [48]. In normal cells, NF-κB combines with IkB in the cytoplasm to form inactive complexes, while excess APAP leads to hepatocyte injury and triggers transcriptional activation of pro-inflammatory factors (IL-1β and TNF-α) in the Kupffer cells [49,50]. These inflammatory substances function as activators, causing the strongest inhibitor, IkB, to be phosphorylated and the trimer to degrade quickly. This makes it possible for *p*-NF-κB to enter the nucleus to trigger the COX-2 signal, escalating the inflammation reaction [51,52]. Studies have shown that the inhibition of liver inflammation reduces APAP-induced hepatotoxicity [53]. Figure 7 displays the expression of several proteins connected with inflammatory processes in the liver. Compared with those in Group C, the proteins *p*-IκBα/IκBα (Figure 7a,b), *p*-NF-κB/NF-κB (Figure 7a,c), and COX-2 (Figure 7a,d) were increased considerably in Group M (*p* < 0.05). The contents of these proteins in Group EH was decreased significantly (*p* < 0.05), although *p*-NF-κB/NF-κB and COX-2 did not improve significantly in Group EL compared with Group M. As presented by the immunohistochemical results (Figure 7e,f), nuclear-nuclear factor κB (N-NF-κB) was expressed in the cell nucleus. Compared with Group C, higher expression of N-NF-κB was observed in Group M. However, the contents of N-NF-κB in both treatment groups (EL and EH), especially the EH group, decreased in comparison Group M (Figure 7e). The results of Western blotting and immunohistochemistry show that NAPQI, an intermediate metabolite of APAP toxicity, activated the Kupffer cells to secrete proinflammatory cytokines and then activated the NF-κB signaling pathway, leading to an increase in COX-2 protein expression, further aggravating the inflammatory response. However, ECS was able to reverse this process. These results suggest that ECS can attenuate the inflammatory reaction in APAP-induced liver damage.

### 3.6. ECS Inhibited the Apoptosis of Hepatocytes in APAP-Induced Liver Injury

It has been found that APAP-induced ALI is related to apoptosis [54]. APAP-induced cell death is caused by massive mitochondrial dysfunction and damage to the nuclear DNA [55]. The PI3K/Akt signaling pathway is a crucial point for hepatoprotection via inhibiting apoptosis under oxidative stress and inflammatory conditions [24]. PI3K is an intracellular phosphatidylinositol kinase, and Akt is one of its key downstream effectors. PI3K generates a secondary messenger (*p*-PI3K) at the plasma membrane to further activate Akt, then the activated Akt enhances the Bcl-2 and inhibits Bax to exert antiapoptotic effects and reduce liver injury [56,57]. Bcl-2 is an important pro-survival protein that can bind to the outer mitochondrial membrane and cause damage, leading to depolarization of the mitochondrial membrane, antagonizing the proapoptotic Bax homodimer, and inhibiting damage to the mitochondrial DNA as well as cell death [54,57]. 

**Figure 8:**
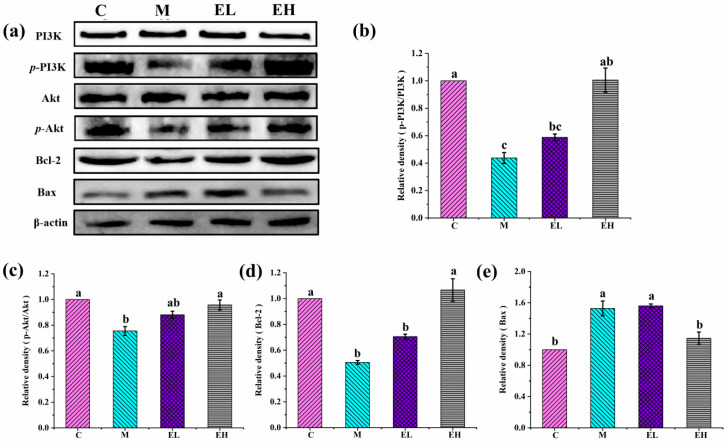
Effects of ECS on the contents of key proteins participating in the apoptotic signaling pathway. (**a**) Immunoblotting of *p*-PI3K/PI3K, *p*-Akt/Akt, Bax, and Bcl-2. (**b**–**e**), illuminating the relative quantification of the *p*-PI3K/PI3K, p-Akt/Akt, Bax, and Bcl-2 proteins, respectively. The relative protein contents were normalized with β-actin and Group C, shown as grayscale. Data are presented as the mean ± S.E. (*n* = 4). For each protein, significant differences are marked by different letters (*p* < 0.05). *p*-PI3K/PI3K, *p*-Akt/Akt, Bax, and Bcl-2 represent phosphorylated-phosphatidylinositol 3-kinase/phosphatidylinositol 3-kinase, phosphorylated-protein kinase B/protein kinase B, Bcl-2-associated X protein, and B-cell lymphoma gene 2, respectively; C, M, EL, and EH represent control, model, low-dose, and high-dose ECS groups (300 or 600 mg kg^−1^ of b.w.), respectively.

**Figure 9:**
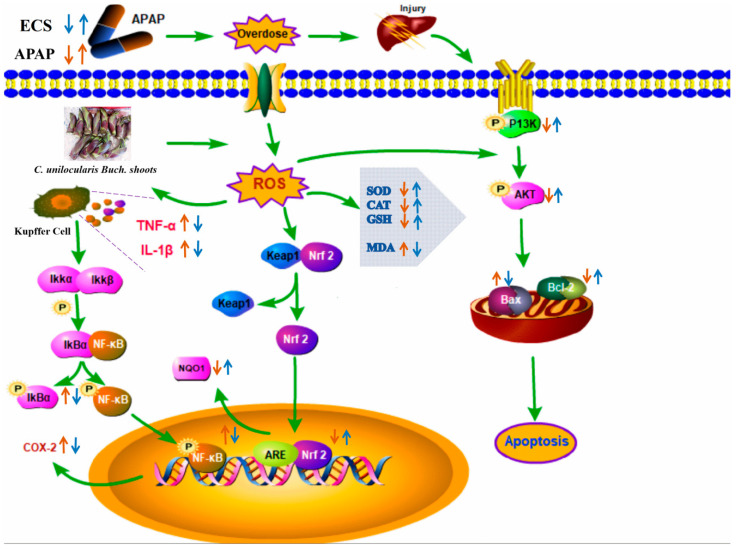
The main potential mechanisms of liver injury caused by APAP in mice with or without the ethanol extract of the wild vegetable shutou. ROS, SOD, CAT, GSH, and MDA represent reactive oxygen species, superoxide dismutase, catalase, reduced glutathione, and malondialdehyde, respectively; *p*-IκBα, *p*-NF-κB, and COX-2 represent phosphorylated-inhibitor kappa B al-pha, phosphorylated-nuclear factor κB, and cyclooxygenase-2, respectively; *p*-PI3K/PI3K, *p*-Akt/Akt, Nrf2, NQO1, TNF-α, IL-1β, Bax, and Bcl-2 represent phosphorylated-phosphatidylinositol 3-kinase/phosphatidylinositol 3-kinase, phosphorylated-protein kinase B/protein kinase B, nuclear factor erythroid-2-related factor 2, NADH quinone oxidoreductase 1, tumor necrosis factor-α, interleukin-1β, Bcl-2-associated X protein, and B-cell lymphoma gene 2, respectively.

According to Figure 8, in comparison with Group C, the contents of *p*-PI3K/PI3K, *p*-Akt/Akt and Bcl-2 were significantly reduced and Bax was greatly upgraded in Group M (*p* < 0.05), which indicates that APAP remarkably suppresses the phosphorylation of PI3K/Akt and disrupts the Bax/Bcl-2 balance. However, after ECS treatment the contents of *p*-PI3K/PI3K, *p*-Akt/Akt, and Bcl-2, improved remarkably, especially in the EH group, and were close to those of Group C (*p* > 0.05). The expression of Bax, especially in the EH group, declined significantly (*p* < 0.05) in comparison with Group M. In the present study, ECS may have increased the levels of *p*-PI3K, *p*-Akt, and Bcl-2 while inhibiting those of Bax. All of this evidence strongly suggests that ECS may inhibit apoptosis by regulating the PI3K/Akt signaling pathway to adjust the expression of Bcl-2 and Bax (Figure 9). A related study showed that natural active ingredients of plant species such as tempol and forsythiaside A could inhibit the apoptosis of hepatocytes by triggering the PI3K/Akt pathway to normalize liver function in APAP-induced acute hepatotoxic mice [22,58], which is consistent with our results. 

## 4. Conclusions

The present work indicates that ECS can protect against ALI caused by APAP in mice. ECS, especially at 600 mg kg^−1^ b.w., reduced three serum signature indicators (ALT, AST, and ALP), which may be due to increased activity of antioxidant enzymes (SOD, CAT, and GSH) in the tissue and the enhanced expression of Nrf2 protein, which increased the expression of NQO1, thereby improving the oxidative stress response caused by excessive APAP uptake. In addition, ECS decreased MDA levels and the content of inflammatory cytokines, including TNF-α and IL-1β, and reduced lipid peroxidation and the inflammatory response. ECS regulated the contents of the corresponding apoptotic proteins (Bax and Bcl-2) by enhancing the PI3K/Akt protein pathway. Moreover, according to our results, ECS may effectively prevent liver injury caused by oxidative stress induced by other factors, which needs to be further investigated in the future. In summary, ECS may have the potential to be exploited as a functional food for preventing liver injury caused by APAP abuse.

## Data Availability

The data that support the findings of this study are available from the corresponding author upon reasonable request.

## Supplementary Materials

The following supporting information can be downloaded at: https://www.mdpi.com/article/10.3390/foods12163109/s1, Figure S1: Fresh sample of the wild vegetable shutou (*Crateva unilocularis* Buch.). Figure S2: The chromatograms of the ethanol extract of the wild vegetable shutou (*Crateva unilocularis* Buch.) peak characterization and the detailed MS data are summarized in Table S1. Table S1. Phytochemicals characterized in the wild vegetable shutou (*Crateva unilocularis* Buch.) by UHPLC-ESI-HRMS/MS with a negative mode.
