# Supplementary material for: The Protective Effect and Mechanism of a Phytochemical Extract from the Wild Vegetable Shutou (Crateva unilocularis Buch.) against Acetaminophen-Induced Liver Injury in Mice
Source: Foods. 2023 Aug 18;12(16):3109. doi: 10.3390/foods12163109 (PMC10453156; doi:10.3390/foods12163109)
Supplement: Supplementary file 1 [file foods-12-03109-s001.zip › foods-2433166-supplementary.pdf]

SUPPORTING INFORMATION

The Protective Effect and Mechanism of a Phytochemical Extract from the Wild Vegetable Shutou (*Crateva unilocularis* Buch.) against Acetaminophen-Induced Liver Injury in Mice

Meimei Shan [1,2], Qian Ma [1,3], Yilin Sun [1,3], Fengyi Gao [2,*] and Shengbao Cai [1,*]

[1] Faculty of Food Science and Engineering, Kunming University of Science and Technology, Kunming 650500, China; 18239031665@163.com (M.S.); kmustma95@163.com (Q.M.); syl115630519@163.com (Y.S.)
[2] College of Biology and Food, Shangqiu Normal University, Shangqiu 476000, China
[3] Department of Food Science, Northeast Agricultural University, Harbin 150030, China
* Correspondence: gaofengyi@sqnu.edu.cn (F.G.); caikmust2013@163.com (S.C.)

Figure S1. Fresh sample of the wild vegetable shutou (*Crateva unilocularis* Buch.)

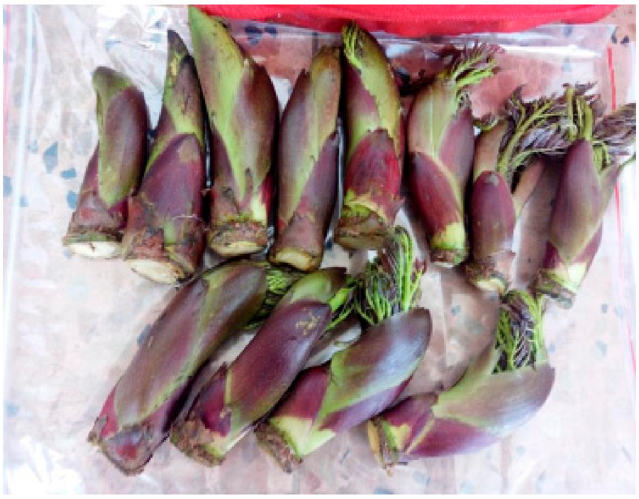

Figure S2. The chromatograms of the ethanol extract of the wild vegetable shutou (*Crateva unilocularis* Buch.) peak identification and their MS data are shown in Table S1.

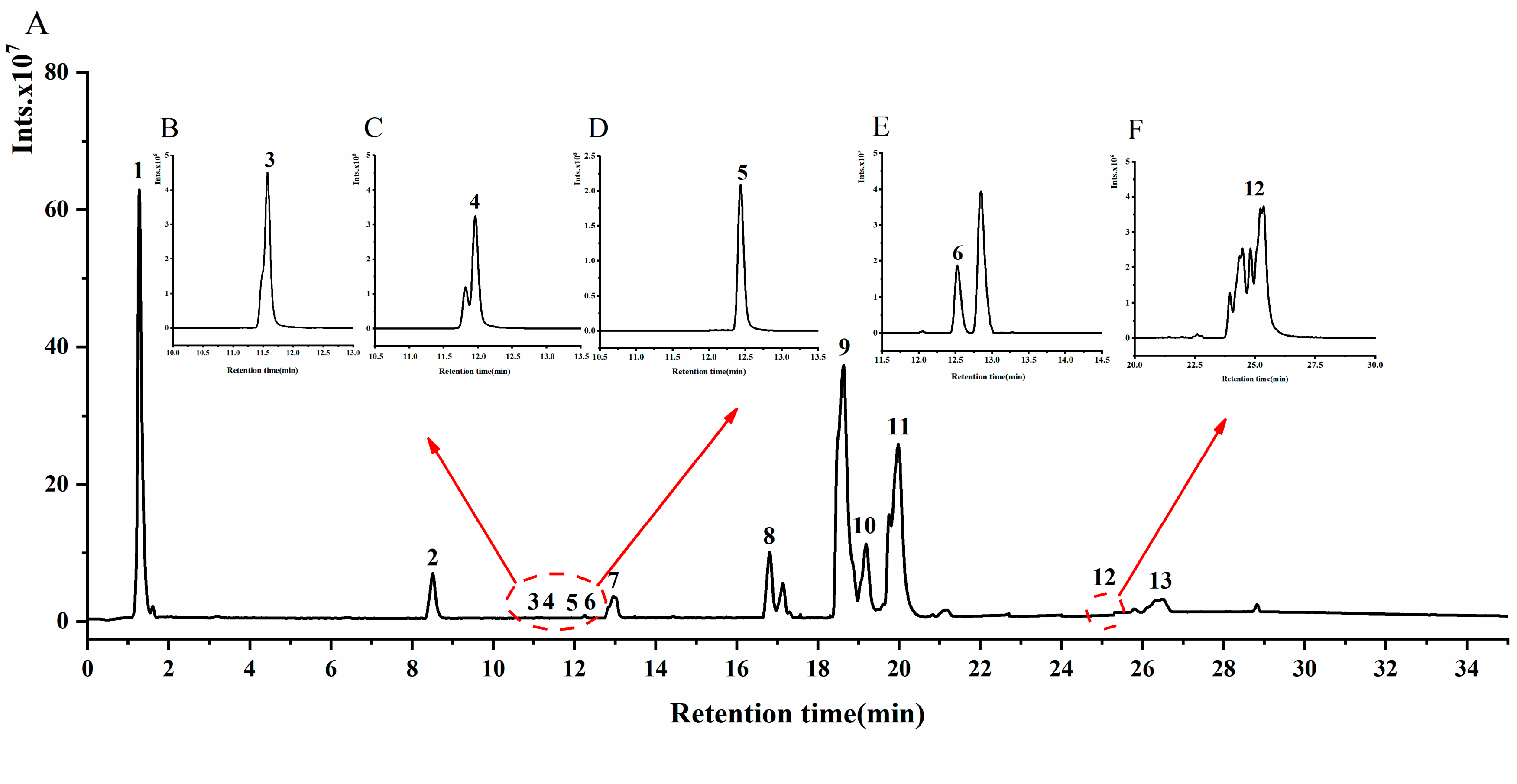

Table S1. Phytochemical compounds identified in the wild vegetable shutou (Crateva unilocularis Buch.) by UHPLC-ESI-HRMS/MS in negative mode.

Peak No.	Compounds	Molecular formula	Retention time (min)	[M− H]- (m/z)	MS/MS Fragment Ions	Dry extract（μg/g）	Reference
1	Quinic acid	$C_7H_{12}O_6$	1.22	191.0557	93.0334(71.37),85.0283(100),67.0176（15.56)	65350.51±2927.70	[26]
2	Chlorogenic acid	$C_{16}H_{18}O_9$	8.54	353.0887	191.0558(100),135.0443(5.61),85.0283(35.12)	11436.08±512.34	[26]
3	Rutin	$C_{27}H_{30}O_{16}$	11.59	609.1479	301.0349(50.02),300.0283(100)	674.87±30.23	[26]
4	Myricetin-3-O-rhamnoside	$C_{21}H_{20}O_{12}$	11.77	463.0895	300.0283(100),271.0255(64.01)	341.92±15.32	[26]
5	Kaempferol-3-O-rutinoside	$C_{27}H_{30}O_{15}$	12.39	593.1530	284.0333(78.50)	76.35±3.42	[26]
6	Luteolin-7-O-glucoside	$C_{21}H_{20}O_{11}$	12.52	447.0940	256.0356(15.88）,227.0352(66.58)	37.74±1.69	[26]
7	3,4-Dicaffeoylquinic acid	$C_{25}H_{24}O_{12}$	13.09	515.1207	191.0558(100),179.0346(64.06),135.0444(32.10)	5260.10±235.65	[26]
8	Achyranthoside D	$C_{53}H_{82}O_{25}$	16,81	1117.5464	1117.5470(100)	25810.04±1156.29	[26]
9	Ginsenoside Ro	$C_{48}H_{76}O_{19}$	18.71	955.4934	793.4399(15.03),731.4374(2.20),455.3560(0.23)	62152.63±4321.32	[26]
10	Chikusetsusaponin IV	$C_{47}H_{74}O_{18}$	19.17	925.4831	793.4398(12.82),455.3530(0.24）	36650.89±1641.96	[26]
11	Chikusetsusaponin IVa	$C_{42}H_{66}O_{14}$	20.00	793.4400	631.3870(29.97),587.3955(1.55),569.3863(10.88, 455.3560(1.79）	54859.01±2457.68	[26]
12	28-Deglucosyl chikusetsusaponin IV	$C_{41}H_{64}O_{13}$	25.18	763.4298	569.3860(6.97),551.3751(2.48),455.3547(4.73)	20.83±0.93	[26]
13	28-Deglucosyl chikusetsusaponin IVa	$C_{36}H_{56}O_9$	26.58	631.3870	455.3552(22.08),113.0234(43.08),85.0283(47.23)	8175.01±366.24	[26]

RT: retention time; Values are expressed as the mean ± S.D. (n = 3, μg/g dry extract); Quinic acid standard was used for quantifying the compounds

1; Chlorogenic acid standard was used for quantifying the compounds 2 and was used for semi-quantifying the compounds 7 ;Rutin standard was used for quantifying the compounds 3；Myricetin-3-O-rhamnoside was used for quantifying the compounds 4；Kaempferol-3-O-rutinoside was used for quantifying the compounds 5；Luteolin-7-O-glucoside was used for quantifying the compounds 6；Achyranthoside D was used for quantifying the compounds8；Ginsenoside Ro was used for quantifying the compounds 9；Chikusetsusaponin IV was used for quantifying the compounds 10；Compounds11 were semi-quantifified with Chikusetsusaponin IV standard;Deglucosyl chikusetsusaponin IV was used for quantifying the compounds 12；Compounds13 were semi-quantifified with 28-Deglucosyl chikusetsusaponin IV standard
